# Ethanol injection therapy for small hepatocellular carcinomas located beneath a large vessel using a curved percutaneous ethanol injection therapy needle

**DOI:** 10.3892/ol.2014.2053

**Published:** 2014-04-09

**Authors:** SEISHIRO WATANABE, ASAHIRO MORISHITA, AKIHIRO DEGUCHI, SEIJI NAKAI, TEPPEI SAKAMOTO, KOJI FUJITA, EMIKO MAEDA, TAKAKO NOMURA, JOJI TANI, HISAAKI MIYOSHI, HIROHITO YONEYAMA, SHINTARO FUJIWARA, HIDEKI KOBARA, HIROHITO MORI, TAKASHI HIMOTO, TSUTOMU MASAKI

**Affiliations:** 1 Department of Internal Medicine, Kagawa Prefectural Central Hospital, Takamatsu, Kagawa 760-8557, Japan; 2 Department of Gastroenterology and Neurology, Kagawa University School of Medicine, Miki-cho, Kida-gun, Kagawa 761-0793, Japan; 3 Department of Integrated Medicine, Kagawa University School of Medicine, Miki-cho, Kida-gun, Kagawa 761-0793, Japan

**Keywords:** curved needle, percutaneous ethanol injection therapy, hepatocellular carcinoma

## Abstract

Percutaneous ethanol injection therapy (PEIT) has been administered as a safe therapeutic modality for patients with small hepatocellular carcinoma (HCC). Due to the nature of the straight approaching line of a PEIT or radiofrequency ablation needle, penetrating the vessels that are interposed between the dermal insertion point and the nodule is unavoidable. A device with an overcoat needle and coaxial curved PEIT needle was created that facilitated a detour around interposing large vessels in order to avoid unnecessary harmful effects that result from the PEIT procedure. Two cases of HCC located adjacent to a neighboring large vessel were treated with a curved PEIT needle. The curved PEIT needle, which is connected to an outer needle, enabled deviation around the interposing vessels and successful connection with the HCC. Careful use of the curved line of the PEIT needle enabled the safe and successful performance of the PEIT without any requirement for specific training. This hand-assisted technique may be an applicable treatment for small HCC located beneath large vessels as a direct therapeutic method using ultrasound guidance.

## Introduction

Techniques for percutaneous ethanol injection therapy (PEIT) have been widely established to treat hepatocellular carcinoma (HCC) and have once been applied in larger size cancer nodules using a multiple-insertion technique (1). With the development of the radiofrequency ablation (RFA) technique, the adoption of PEIT became limited to small-sized HCC nodules or those locating in the vascular-rich portions of the liver, including the hepatic hilus (2). Small early-stage HCC have been detected frequently by periodic surveillance of cirrhotic patients (3). Among these cases, there were certain cases where the PEIT needle unavoidably penetrated the large vessels between the cancer nodules during PEIT. For the treatment of these special cases, a curved PEIT needle was created to avoid the unnecessary adverse effects that are associated with this treatment modality.

## Patients and methods

### Patient 1

A 79-year-old male, exhibiting a HCC that was 2.6 cm in diameter and located in Couinaud’s S4 subsegment (S4), underwent conventional PEIT with a mixture of 5 ml of 99.5% ethanol and 0.6 ml of iodized oil (Lipiodol) in January 2005 (4). The Lipiodol mixture method was adopted to improve the visibility of the HCC nodule during computed tomography (CT) scanning (5). The patient was treated twice in 2001 with RFA for HCCs that were 1.5 and 2.5 cm in diameter, and located in S2 and S8, respectively. The histology of a biopsy specimen obtained at that time showed a well-differentiated HCC that was positive for the hepatitis C virus and exhibited liver cirrhosis.

**Figure 1:**
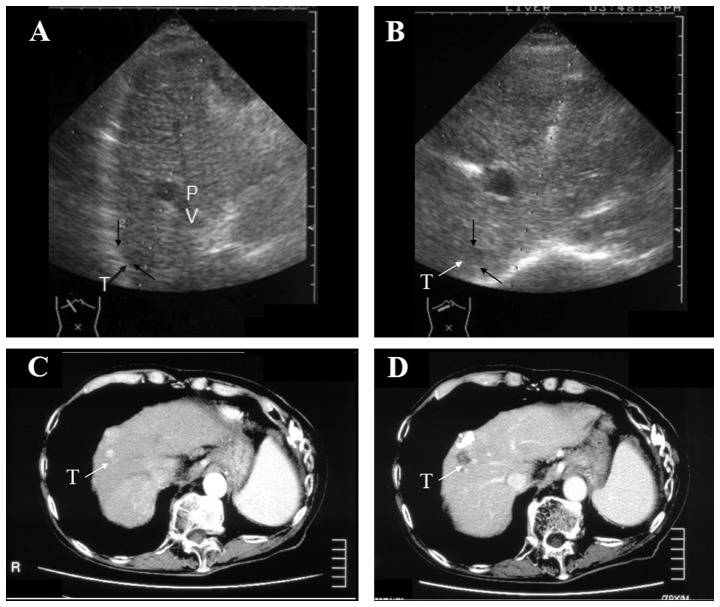
Image findings for patient 1. (A) Ultrasonograms captured by right intercostal scanning prior to therapy. A large portal vein (PV) intersects the puncture point and the nodule (arrow). A curved percutaneous ethanol injection therapy (PEIT) needle was used to conduct the right subcostal scanning during therapy. (B) The needle tract of an overcoat needle is observed at the left side of the right PV. (C) A small contrast enhanced tumor (1.6×1.6 cm) is located in S4 in the arterial phase of an abdominal, dynamic computed tomography image in patient 1 prior to therapy I (arrow). (D) A white colored nodule on the subcapsular region of the liver demonstrates the remaining iodized oil (Lipiodol) that accumulated during the previous PEIT (with a mixture of ethanol and Lipiodol) in 2005. Subsequent to the therapy, the tumor evolved into a low-density area without contrast enhancement (arrow).

In January 2005, an additional HCC (size, 1.6×1.6 cm) was identified behind the treated HCC, which was located just beneath a branch of the large left portal vein on the approaching line of the ultrasound image (Fig. 1A). A curved PEIT needle was used for the treatment of this HCC.

### Patient 2

**Figure 2:**
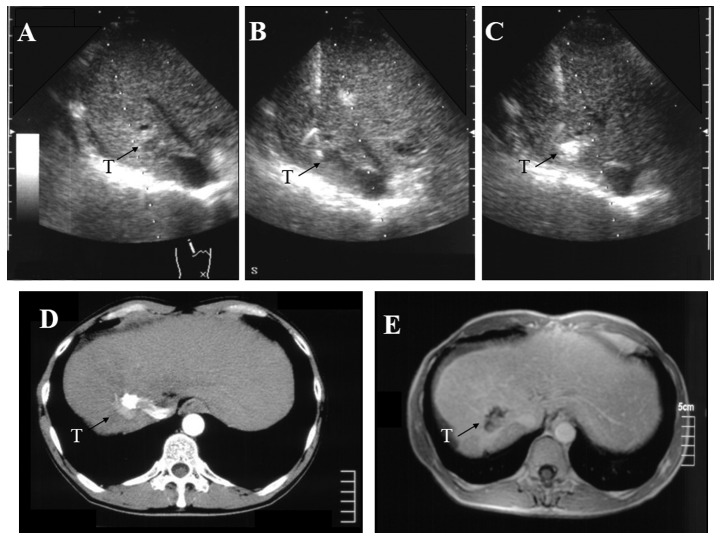
Image findings for patient 2. (A) Ultrasonograms captured by right intercostal scanning prior to therapy. A right portal vein (PV) intersects the puncture point and a low-echoic nodule prior to therapy (arrow). (B) A needle tract of an overcoat needle and an extended curved percutaneous ethanol injection therapy (PEIT) needle from the overcoat needle are observed at the right side of the right PV. A curved PEIT needle deviates around the vessel to the cancer nodule and is inserted in the right side of the tumor (arrow). (C) Immediately following the ethanol injection, the tumor area undergoes a hyperechoic change (arrow). (D) A small enhanced nodule (1.3×1.3 cm) is located in S7 in the arterial phase of an abdominal dynamic computed tomography image prior to therapy I (arrow). (E) Arterial phase image of the abdominal dynamic magnetic resonance imaging scan that was obtained subsequent to the therapy using a curved PEIT needle. The treated area evolved into a low-intensity area following therapy (arrow).

A 56-year-old male who was positive for the hepatitis C virus and exhibited liver cirrhosis was referred to the Kagawa University Hospital (Miki-cho, Japan) for the treatment of recurrent HCC. In 2000, the patient was treated with RFA twice for HCCs that were 2.0 and 1.6 cm in diameter, and located in S8 and S7, respectively. An additional HCC, 1.9 cm in diameter, was identified in S7 and treated angiographically using a mixture of Lipiodol and a lipophilic anticancer agent, styrene maleic acid neocarzinostatin (Lipiodol; Yamanouchi Pharmaceutical Co., Ltd., Tokyo, Japan), which was followed by a transcatheter embolization of the tumor (6) in February 2005. Three months later, another tumor (diameter, 1.3 cm) was observed in S7 on the posterior side of the branch of the right portal vein (Fig. 2A). This portal vein was located just above the tumor, and a conventional straight PEIT needle would intersect the puncture point of the tumor on the ultrasound image. This HCC was treated using a curved PEIT needle. As the patient’s renal function was low, dynamic magnetic resonance imaging (MRI) was employed to evaluate the therapeutic effects.

### Procedure

Prior to performing the procedure, written informed consent was obtained from each patient’s family and the study was approved by the clinal ethics committee of Kagawa University Hospital (Kagawa, Japan). The patients were treated with the same method as follows.

**Figure 3:**
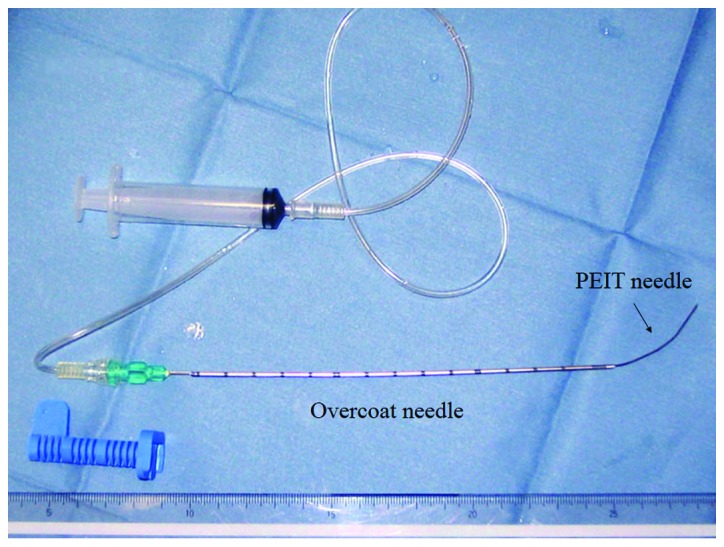
A curved percutaneous ethanol injection therapy (PEIT) needle that is connected to a syringe via an extension tube, is coaxially prepared in an overcoat needle. Note that the head of the PEIT needle is curved in a fishhook shape.

An 18-gauge PEIT needle (20-cm long; Hakko Co., Ltd., Chikuma, Japan), which was manually curved into a fishhook shape, was prepared (Fig. 3). A curved portion of the 18-gauge needle was pushed inside the straight 16-gauge overcoat needle (shortened to a 12-cm length; Bard Biopty-Cut Needle, Discovery Bay, CA, USA). Prior to commencing the hand-assisted maneuver, the curved PEIT needle was drawn back into the overcoat needle. The procedure was guided via the ultrasound monitor to assess whether the curved PEIT needle, which extended from the overcoat needle, approached the cancer nodule.

Subsequent to receiving local anesthetic, the coaxially prepared needles were held by the overcoat needle and inserted with ultrasonic guidance into the lateral side of the interposing vessel edge, above the cancer nodule. The curved PEIT needle was extended slowly from the overcoat needle towards the HCC. The quantity of ethanol that was injected by a single shot was 0.5–1.0 ml. A total of 5 ml of 99.5% ethanol was injected in small doses through the curved PEIT needle.

## Results

A large portal vein intersected the dermal insertion point of the PEIT needle and the cancer nodule in the two patients. To reinforce the straight structure of the outer needle following insertion of the inner curved PEIT needle, a 16-gauge outer needle was required. As the curved PEIT needle was extended from the overcoat needle into the parenchyma of the liver at the lateral side of a large vessel neighboring the HCC, the needle did not maintain the original curve that was previously visualized. The curved PEIT needle gradually lost its shape and weakened in the liver parenchyma, resulting in the angle of the anticipating approach curve becoming a weak curved line in the fibrotic liver. Therefore, it was required that the inner PEIT needle was fixed as a stronger curve in advance, or that an overcoat needle was inserted nearer to the target than the simulation line. As a result of the curve in the needle, it detoured the large vessel adjacent to the HCC (Figs. 1B and 2B). Immediately following the ethanol injection, the tumor area became increasingly hyperechoic (Fig. 2C). The arterial phase of an abdominal dynamic CT image in patient 1 prior to and following the therapy is shown in Fig. 1C and D. The treated HCC evolved into a low-density area without contrast enhancement. The arterial phase of an abdominal dynamic CT image in patient 2 prior to therapy is shown in Fig. 2D. Compared with the HCC of the prior treatment, the tumor evolved into a low-intensity area without enhancement as shown in the abdominal dynamic MRI image following therapy (Fig. 2E). The two patients were successfully treated with this method without any specific training required for performing the procedure.

## Discussion

Ultrasound-guided percutaneous ablation therapy has developed during the past two decades (7). Prior to the introduction of RFA therapy, PEIT was the most effective method for the initial treatment of patients with well-differentiated HCC (tumor size, <15 mm in greatest dimension) (8). Duplex color Doppler ultrasound is effective for identifying tortuous vessels that supply HCCs in the cirrhotic liver. Previously, PEIT was performed for palliative ablation of tumors supplying vessels in the HCC nodule and to minimize the tumor vascularization using duplex color Doppler ultrasound (9).

In multivariate analysis, the significant prognostic factors have been determined as local recurrence, and tumor size and number. This indicates that successfully attaining a complete treatment for HCC during the first treatment is significant for improving the prognosis of patients with HCC (10). RFA exhibits superior therapeutic results compared with PEIT. The overall survival rate was higher in patients treated with RFA compared with those that were treated with PEIT. Furthermore, the local recurrence rate is higher in patients treated with PEIT compared with those treated with RFA (11). RFA has been widely investigated as an alternative to PEIT, however, due to its mild invasiveness to the patient, PEIT retained its validity for the treatment of small HCC. PEIT is widely used for encapsulated small tumors in livers (12,13). PEIT can also be considered as a treatment of choice for small HCCs, particularly in patients with poor liver reserve or comorbidity that make them potentially poor surgical candidates (14,15). In addition, PEIT is a useful alternative where RFA is unavailable (16). Repeated PEIT is permitted in patients with an adequate liver function. In addition, during follow-up the intrahepatic recurrence of HCC is the predominant factor that affects survival rate (17). The prognosis of patients with HCC who undergo incurative therapy is extremely poor.

Furthermore, for patients with small HCC, PEIT may produce a survival rate comparable to surgical resection (18,19). By contrast, a previous study on hepatic resection showed higher survival rates compared with non-surgical therapies in small HCC. In clinical stage I cases with a solitary tumor <2 cm in diameter, in all clinical stages with a solitary tumor >2 cm and in the clinical stage II cases with two tumors >2 cm, the hepatic resection showed higher survival rates compared with the non-surgical groups (20).

PEIT-associated adverse effects are not as serious compared with those of RFA therapies, however, studies regarding vascular or bile duct damage, such as hepatic infarction (21) or portal branch venous thrombosis (22), have been reported. Since complications include acute and delayed vascular injury following ethanol injection, patients require a long period of follow-up subsequent to treatment (23). To avoid adverse effects, PEIT may be useful in those patients with small HCC where taking a straight approach line, guided by ultrasound imaging, is difficult due to intervening vessels between the puncture point and the HCC. A study by Zuo *et al* (24) also reported that CT-guided PEIT with a curved needle is effective for the treatment of malignant liver neoplasms, which strongly supports the use of a novel ultrasound-guided PEIT with a curved needle in the present study. Although the procedure is not simple, it is feasible for physicians proficient in performing the regular PEIT technique.

In conclusion, the present study reports two cases of HCC treated with a curved PEIT needle. For the purpose of achieving PEIT safely, a novel PEIT needle was created with an overcoat needle and a coaxial curved PEIT needle for the treatment of small HCC adjacent to an intrahepatic large vessel. In cases of patients with small HCC, which is difficult to approach with a conventional strait PEIT needle, the curved PEIT needle presented in the current study may be effective in avoiding unnecessary adverse effects
